# Predictor and subgroup analysis of somatic symptoms and emotional exhaustion among university students in Germany during the SARS-CoV-2 pandemic: a longitudinal analysis

**DOI:** 10.3389/fpubh.2026.1827225

**Published:** 2026-05-13

**Authors:** Katharina Wörle, Pavel Dietz, Stephan Letzel, Thomas Rigotti, Lina M. Mülder, Jennifer L. Reichel

**Affiliations:** 1 Institute of Occupational, Social and Environmental Medicine, University Medical Center of the Johannes Gutenberg University, Mainz, Germany; 2 Institute for Teachers' Health at the Institute of Occupational, Social and Environmental Medicine, University Medical Center of the Johannes Gutenberg University, Mainz, Germany; 3 Department of Work, Organizational, and Business Psychology, Institute for Psychology, Johannes Gutenberg University, Mainz, Germany; 4 Leibniz Institute for Resilience Research, Mainz, Germany; 5 Department of Psychology, Institute of Mind, Brain, and Behavior, HMU Health and Medical University, Potsdam, Germany; 6 Corporate Health Management, Human Resources and Legal Affairs, Technical University of Darmstadt, Darmstadt, Germany

**Keywords:** academic demands, academic resources, COVID-19, resilience, tertiary education

## Abstract

**Introduction:**

The mental and physical health of university students is not just of interest to the students themselves, but to our whole society. While most research has focused on medical students, other disciplines remain underexplored. Therefore, this study aims (1) to assess the prevalence and development of emotional exhaustion and somatic symptoms from 2020 to 2021 in both cross-sectional and longitudinal samples of university students, (2) to examine subgroup differences based on demographics and study fields, and (3) to investigate predictors of symptom changes.

**Methods:**

Data were collected from students from a large university in Germany, using two surveys conducted 1 year apart. Independent-samples *t*-tests were used to examine subgroup differences, and multiple regression analyses to identify predictors of changes in symptoms.

**Results:**

A total of 3,066 students participated in the 2020 survey, and 1,438 in 2021, with a longitudinal sample of *N* = 264. Approximately one-third of all students exhibited critical somatic symptom scores, and 18%−19% reached high emotional exhaustion scores. Non-medical students reported higher somatic symptoms compared to medical students, and females as well as first-year students showed higher levels of both emotional exhaustion and somatic symptoms. Longitudinal analyses indicated that emotional exhaustion increased with higher perceived work complexity.

**Discussion:**

These findings highlight considerable mental and physical health impairments not only among medical students but also across diverse study fields, with particular vulnerabilities among women and new students. Moreover, the link between emotional exhaustion and work complexity underscores academic pressure as a key contributor to students' mental health. Thus, universities could set the base for an efficient future workforce with an early health promoting education, preventive measures addressing work complexity and targeted support to vulnerable groups.

## Introduction

Universities might play an important role in the prevention and occurrence of mental health problems in future professionals (1–3). According to Sheldon et al. (2021) (4), university students worldwide are exposed to a wide range of factors affecting their mental well-being. Since overall well-being includes both mental and physical health (5), the present study considers both components.

Previous research showed that university students in general seem to be susceptible to burnout (6). Focusing on the German population, Olson et al. (2023) (7) stated that “Burnout is a major problem in higher education setting, with almost one third of the students being affected.” (p.7). Existing research suggested that academic burnout might start with emotional exhaustion, which therefore, could be the first symptom to appear (8). Emotional exhaustion is defined as a state of feeling emotionally drained and overwhelmed (9). According to a meta-analysis including data from 24 studies and 17,431 medical students worldwide, 40.8% of medical students are affected from emotional exhaustion (10). Obregon et al. (2020) (11) utilized a cut-off value to reveal high emotional exhaustion scores and therefore signs of severe burnout. The same study demonstrated that 8% of medical students exceeded the cut-off value, indicating high emotional exhaustion scores (11).

Despite being an equivalent component of well-being, physical changes in university students are largely overlooked (5, 12). Physical manifestations such as headaches, sleep disturbances and different kinds of pain can be summed up as somatic symptoms (13). Distressing somatic symptoms, regardless of whether they have a medical explanation, are linked to severe psychological distress and numerous mental disorders (14, 15). Additionally, studies of the past proposed that the development of somatic symptoms could be indirectly influenced by everyday stressors (16, 17). If somatic symptoms persist and are accompanied by excessive thoughts, feelings, or behaviors, they are classified as somatic symptom disorder according to DSM-V (18, 19). A high prevalence of somatic symptom disorders ranging from 26.2 to 34.8% among patients consulting primary care physicians was reported in a meta-analysis of 32 studies (20). It is one of the most common problems general practitioners are confronted with and therefore a major concern in the general population (21). It has previously been observed that 72.3% of Czech university students and 69.5% of Slovak university students suffered from somatic symptoms (22). Despite the existing evidence suggesting that somatic symptoms have become a prominent issue among university students, research remains limited and focuses particularly on medical students. Several studies proposed that medical students might be more affected by somatic symptoms compared to the general population and therefore, present as a risk group (23, 24). Medical students in a variety of countries exhibit high rates of somatic symptoms, which are strongly associated with psychological distress and other mental health issues (12, 13, 23). For example, 50.7% of medical students and 63.6% of dental students at a German University, experienced distressing somatic symptoms (12).

In late 2019, the SARS-CoV-2 virus emerged and led to a global health crisis (25). Implemented protective measures relied on isolation and social distancing in form of widespread lockdowns, impacting people's daily life, work and social interactions worldwide (26, 27). Universities were shut down and internet-based learning became the new form of higher education (28–30). As a result, students faced shifts in their daily routines and study habits, requiring them to adjust to the new learning resources and study spaces (31, 32). Consequently, more students opted to studying alone rather than participating in study groups (31), while also changing their time spent studying (30). However, students faced more than just the impact of university measures: in times of persisting health concerns due to the virus, job loss, missed job offers and internships all compounded by an uncertain environment and future, numerous studies highlight the pandemic's adverse effects on students' psychological well-being (25, 30, 31, 33–37). Conversely, the pandemic-related circumstances also had positive impacts on students (31, 32, 38). For example, some students spent more time with their family and adjusted their exercise and sleep routines (32, 38). Various studies questioned the effect of lockdowns on students' mental health (27, 39, 40). Cross-sectional studies during this period suggested that somatic symptoms were highly prevalent, especially among isolated students (39, 41). However, a previous longitudinal study conducted among 443 students at the University of Mainz, Germany, indicated that the actual change in frequencies of somatic symptoms might be less substantial than anticipated (28). In association with the transfer to online learning, several studies revealed that a heightened use of virtual media resulted in technology-related strain and increased emotional exhaustion (6, 33, 42). Moreover, Olson et al. (2023) (7) underlined that an increase in hours spent studying was associated with a higher risk of emotional exhaustion. In contrast, a prospective observational study involving medical students revealed no significant deterioration in burnout, raising questions about the potential impact on mental health during the SARS-CoV-2 pandemic (43).

From a public health point of view, the insufficient research (12) on somatic symptoms, combined with diagnostic difficulties and delayed recognition (44) are concerning, because impairing somatic symptoms place an enormous burden on patients, general practitioners, the health care system, and therefore society (44–46). A previous study stated that patients suffering from persistent and distressing somatic symptoms frequently seek medical care, leading to twice the annual medical care costs compared to other patients (46). Further studies reported that affected patients experience severe life restrictions and reduction of work participation without receiving the essential mental health support (12, 44, 45). Furthermore, from a societal perspective, it is crucial to take the immense impact of emotional exhaustion on both individuals and society into account (37). In a study conducted in five European dental schools, emotional exhaustion was reported as a risk indicator among university students, predicting future professional burnout (2). Consequently, it not only poses a threat to the quality of education and patient safety, but it also increases the risk for various psychological and physical symptoms, substance abuse, depression and medical illness (3).

It is essential to investigate the prevalence, possible risk factors and vulnerable student groups for emotional exhaustion and somatic symptoms, since these students will shape our future workforce and both the students themselves and society at large, will bear the long-term consequences (6). The existing research on somatic symptoms among university students is insufficient and focuses primarily on students of the healthcare field (12). Among the sparse research that investigated somatic symptoms among diverse study fields, Feussner et al. (2022) (12) and Gavurova et al. (2022) (22) reported a higher prevalence of somatic symptoms among students majoring in Informatics, Mathematics, Information and Communication Technologies and Dentistry compared to medical students. These findings indicate the necessity of including students from diverse fields, not only medicine, in future research regarding somatic symptoms. Previous studies investigating gender differences reported consistent results on females being more vulnerable to somatic symptoms than males (12, 22, 23, 41, 47, 48). There is a growing body of literature that recognizes the importance of emotional exhaustion and burnout among medical students, but far too little comparative research involving other student populations exists, again leaving those of other study fields underrepresented (49). Moreover, previous studies on gender differences (7, 10, 34) and the first academic year (7, 34, 49) are inconsistent and require further investigations. According to the Study Demands-Resources (SD-R) Theory, the study environment has a profound impact on the students' behavior mental health and the efficacy of their learning (50). Aspects of the study environment can be differentiated between demands that require effort (51), such as workload and work complexity (52, 53) and resources providing motivation and support to better manage academic demands (54, 55). These can be categorized into personal resources, such as academic self-efficacy and study resources, such as social support (50). Since the implications for interventions may differ for students' mental and physical health, identifying the demands and resources in their study environment that predict emotional exhaustion and somatic symptoms is key to establish targeted support strategies. While some studies examined potential study-related predictors of somatic symptoms and emotional exhaustion, such as semester progression (7), time spent studying (7), study satisfaction (7), perceived stress (56), social support (56), personality traits (56), and negative stress beliefs (57), they are largely based on cross-sectional data and predominantly focus on students in healthcare-related fields. Therefore, more research is required to determine possible study-related predictors of emotional exhaustion and somatic symptoms. Our study contributes to the present literature by investigating predictors based on the SD-R theory in a longitudinal design. Moreover, in response to the claim of Feussner et al. (2022) (12) that somatic symptoms remain under-researched, our study examined both the mental and physical aspects of student's well-being. Following the claim of Olson et al. (2023) (7) that most research focused on health-care related fields and other disciplines remain underexplored, our study included students from a variety of academic disciplines and even compared medical and dental students to those from other study fields.

In conclusion, the present study aimed (1) to investigate the prevalence and development over time of somatic symptoms and emotional exhaustion among university students from 2020 to 2021, unsing cross-sectional as well as longitudinal data and (2) to explore subgroup differences with regard to sociodemographic and study-related variables (e.g., medical vs. non-medical students, semester progression and gender). Additionally, we aimed (3) to explore potential study-related predictors of somatic symptoms and emotional exhaustion among university students, such as academic self-efficacy, social support from peers and lecturers and academic demands.

## Methods

### Study design and survey procedure

As part of the Healthy Campus Mainz project, two online health surveys were performed among university students at the University of Mainz, Germany in 2020 (T1) and 2021 (T2). All students enrolled at the university were invited to participate in the surveys using the university's central mail list. Using individualized, anonymous codes, the outcomes of participants who took part in both studies could be compared within a longitudinal sample allowing both, cross-sectional as well as longitudinal analysis. Reminder emails were sent several times, and the survey was further promoted through various channels. Participation was anonymous, voluntary and students had to provide informed consent before participation. The surveys were conducted using the Unipark software and contained numerous questions regarding sociodemographic data, health status, health behavior and a variety of other possible factors that could affect student's health. Additionally, specific questions about COVID-19 were covered, including psychosocial stress and pandemic-related worries. Reichel et al. (2021) (58) provide more thorough details about the survey process and the survey's content. Ethical clearance was obtained from the Ethical Committee of the Institute of Psychology of Johannes Gutenberg University Mainz (study I: application-number: 2020-JGU-psychEK-S008) (study II: No. 2021-JGU-psychEK-S017) before starting this study. The research was conducted in compliance with the World Medical Association's Code of Ethics (Declaration of Helsinki) for human experimentation and the American Psychological Association's (APA) Ethical Principles and Guidelines for the Protection of Human Subjects of Research.

### Measures

#### Dependent variables

The burden of somatic symptoms was evaluated using the Somatic Symptom Scale (SSS-8) (59). Participants were asked to rate the extent to which they were bothered by eight somatic symptoms (e.g., stomach or bowel problems, headaches, dizziness), using a 6-point Likert scale ranging from *not at all (0)* to *very much* *(**5**)*. This scale's Cronbach‘s α was 0.74 at T1 and 0.75 at T2. Scores above or equal to 12 (sum score between 0 and 32) (59) indicated a high burden of somatic symptoms. The mean somatic symptoms score was used for correlation, frequencies, subgroup and regression analyses. Therefore, each student could score a maximum of five points.

Five items from the German version of the Maslach Burnout Inventory for students (60) were used to measure emotional exhaustion. Utilizing a 7-point Likert scale, students evaluated five items (e.g., “I feel emotionally drained by my studies”) from *never* *(**1**)* to *always* *(**7**)*. This scale's Cronbach‘s α was 0.91 at T1 and 0.92 at T2. To investigate the prevalence of students with high emotional exhaustion scores, the cutoff value of one standard deviation above the mean was evaluated. Scores above the cutoff value are described as indicators for severe burnout (11). The mean score of emotional exhaustion was used for all analyses and therefore each student could score up to seven points.

#### Independent variables

Regarding sociodemographic data, gender was categorized as male, female, diverse, or open. Among the study-related variables, field of study was classified as either non-medical or medical, with the latter including medicine and dentistry. The variable semester was dichotomized at the second semester, resulting in two groups: first-year students and non-first-year students. Further information on the evaluation of the investigated potential predictors academic-self-efficacy, social support from peers and lecturers, workload and work complexity can be found in Reichel et al. (2021) (58).

### Data analysis

To conduct the statistical analysis, IBM SPSS 27 was used. Descriptive statistics for non-continuous scaled variables are reported as numbers and percentages, while continuous scaled variables are demonstrated as means with standard deviations (SD). Using the cross-sectional sample at T1, independent-samples *t*-tests were performed individually for somatic symptoms and emotional exhaustion to examine subgroup differences regarding sociodemographic (gender) and study-related variables (field of study, semester progression). Only cases with valid responses were included in the analyses.

Correlation analyses were conducted to determine potential study-related predictors. Separate confirmatory factor analyses for our predictor and outcome variables were performed to validate the factorial structure of the variables. Following recommendations by recent guidelines (61–63), the chi-square value (χ^2^), the root mean square error of approximation (RMSEA), the standardized root mean square residual (SRMR), and the comparative fit index (CFI) were used to compare the models. The five-factor model, distinguishing between somatic symptoms, emotional exhaustion, social support from lecturers and peers, academic self-efficacy, workload, and work complexity, and showed the best model fit: $\chi_{314}^{2}$ = 1,056.274, *CFI* = 0.776, *RMSEA* = 0.096 at T1 and $\chi_{314}^{2}$ = 998.059, *CFI* = 0.809, *RMSEA* = 0.094 at T2. The one-factor model ($\chi_{324}^{2}$ = 1,843.400, *CFI* = 0.542, *TLI* = 0.504, *RMSEA* = 0.135 at T1 and $\chi_{324}^{2}$ = 2,073.779, *CFI* = 0.511, *RMSEA* = 0.148 at T2), the two-factor model consisting of one factor including the two outcomes somatic symptoms and emotional exhaustion, and the second factor including the independent variables ($\chi_{323}^{2}$ = 1,617.367, *CFI* = 0.610, *RMSEA* = 0.125 at T1 and $\chi_{323}^{2}$ = 1,722.935, *CFI* = 0.609, *RMSEA* = 0.132 at T2), and the four-dimensional factor solution, combining the two outcomes somatic symptoms and emotional exhaustion into one factor and dividing the independent variables into separate factors ($\chi_{318}^{2}$ = 1,218.793, *CFI* = 0.728, *RMSEA* = 0.105 at T1 and $\chi_{318}^{2}$ = 1,221.650, *CFI* = 0.748, *RMSEA* = 0.107 at T2) showed lower fit indices. The five-factor model was superior to the four-factor model according to significant scaled chi-square differences (T1: Δχ^2^ =162.52, Δ*df* =4, *p* < 0.001; T2: Δ χ^2^ =223.59, Δ*df* =4, *p*<*0.0*01). Therefore, our results confirmed the existence of the five factors (see Supplementary Table 2).

Multiple linear regression analyses were performed to analyze the effect of independent variables on changes in somatic symptoms and emotional exhaustion, including statistically significant variables as possible predictors. The variance inflation factor (VIF) was evaluated, and multicollinearity were examined. By plotting the unstandardized predicted value against the studentized residual in a scatterplot, homoscedasticity of the residuals was tested. Furthermore, normality of residuals was evaluated using a histogram and a P-P plot, and autocorrelation was assessed using the Durbin-Watson statistic. The prediction accuracy was increased by including the autoregressive effect of the variables somatic symptoms and emotional exhaustion at T1 as predictors.

## Results

In total, 3,066 students participated in the online survey in 2020 (T1), 1,438 in 2021 (T2), and 264 at both time-points, building the longitudinal sample. At baseline, the mean age of the longitudinal sample was 22.8 years (SD = 3.4 years), and the majority (82.2%) of participants were female (*n* = 217). The sample's mean age was lower than the mean age of the entire student body at the University of Mainz (24.7). Additionally, women were overrepresented by 23%. Among the participants, 28.9% were 1-year students (*n* = 76) and 14% were enrolled in human medicine or dentistry and therefore, regarded as medical students (*n* = 37). The longitudinal sample differs from the cross-sectional sample of 2020 in terms of age-related variables. This variation in age and year of study is expected, since younger students are more likely to participate in both surveys. Furthermore, the longitudinal sample included a larger percentage of females and medical students compared to the cross-sectional sample. The characteristics of the study samples at each time point are presented in Table 1.

**Table 1:** Characteristics of the study samples.

Characteristic	Longitudinal sample 2020–2021 (*N* = 264)	Cross-sectional sample 2020 (*N* = 3,066)	Cross-sectional sample 2021 (*N* = 1,438)
**Gender** *n* (**%**)	(*n* = 264)	(*n* = 3,066)	(*n* = 1,436)
Female	217 (82.2)	2,225 (72.6)	1,065 (74.2)
Male	46 (17.4)	821 (26.8)	338 (23.5)
Diverse or open	1 (0.4)	20 (0.7)	33 (2.3)
**Age**, years (mean ±*SD*)	18–45 (22.8 ± 3.4)	14–68 (23.4 ± 4.4)	15–69 (23.7 ± 4.7)
**Year of study** *n* (**%**)	(*n* = 263)	(*n* = 3,034)	(*n* = 1,427)
Non-first-year	187 (71.1)	2,264 (74.6)	1,088 (76.2)
First-year	76 (28.9)	770 (25.4)	339 (23.8)
**Field of study** *n* (**%**)	(*n* = 264)	(*n* = 3,012)	(*n* = 1,430)
Non-medical students	227 (86.0)	2,671 (88.7)	1,219 (85.2)
Medical students^1^	37 (14.0)	341 (11.3)	211 (14.8)

SD, standard deviation; Medical students, human medicine and dentistry.

### Prevalence of somatic symptoms and emotional exhaustion of the cross-sectional and longitudinal samples during the SARS-CoV-2 pandemic and the development over time

**Figure 1:**
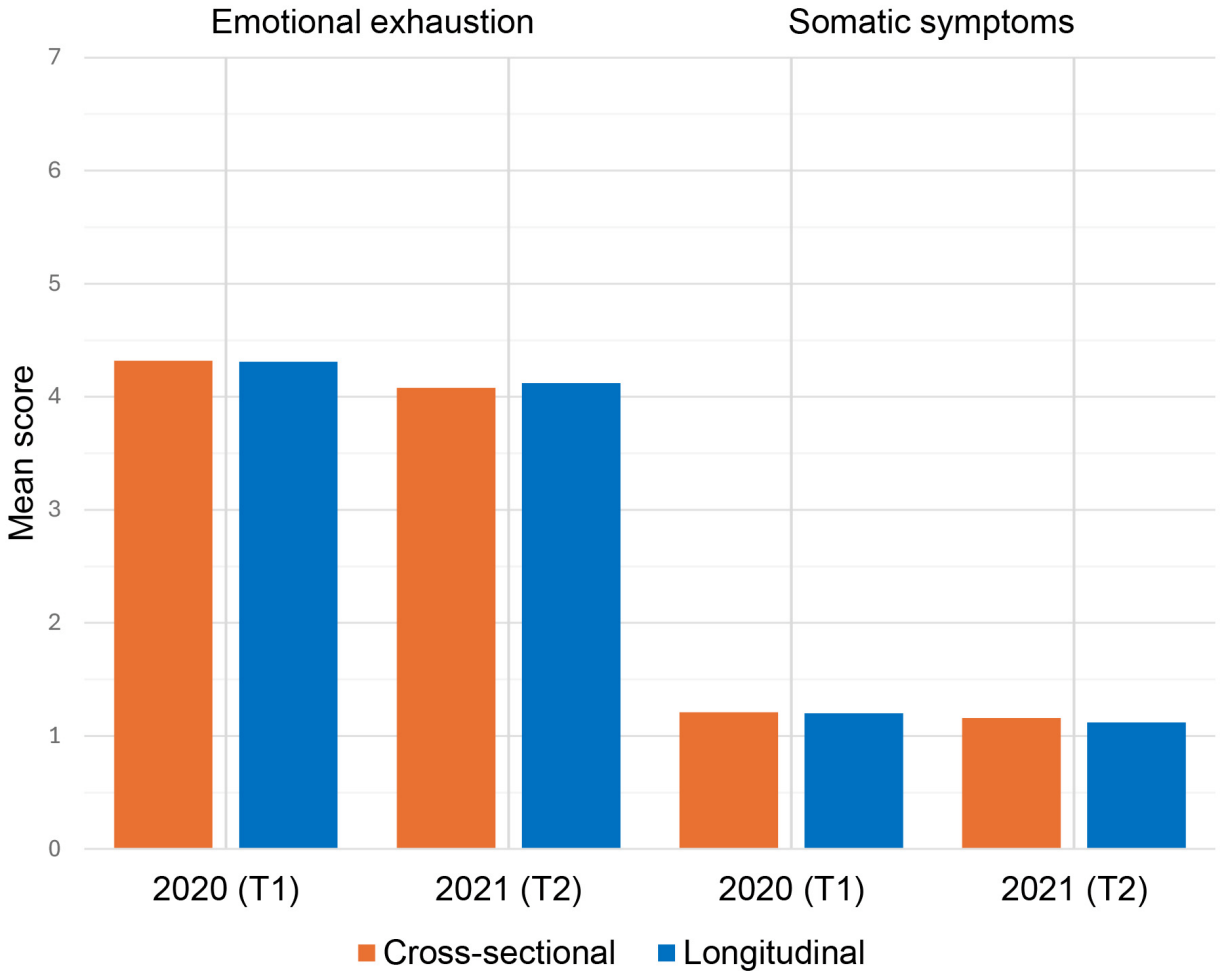
Mean scores for somatic symptoms and emotional exhaustion of the cross-sectional samples (*n*_*somaticsymptoms*_ = 2,892, *n*_*emotionalexhaustion*_ = 2,900 in 2020; *n*_*somaticsymptoms*_ = 1,326, *n*_*emotionalexhaustion*_ = 1,265 in 2021) and the longitudinal study sample (*n* = 258).

In the longitudinal sample, a mean of 1.20 (SD = 0.66) was reported at baseline and a mean of 1.12 (SD = 0.63) in the follow-up survey (see Figure 1). 39.1% exceeded the 12-point cutoff at baseline, compared to 30.8% in the follow-up survey. Given the large sample sizes, the prevalence rates in the cross-sectional samples were also of interest and therefore included. In the cross-sectional samples, the mean score for somatic symptoms was 1.21 (SD = 0.73) in 2020 and 1.16 (SD = 0.69) in 2021. 34.0% of the cross-sectional sample in 2020 and 32.4% in 2021 reported somatic symptom scores greater than or equal to the cutoff value of 12.

Regarding emotional exhaustion, a mean of 4.31 (SD = 1.57) was described in the longitudinal sample at baseline compared to a mean of 4.12 (SD = 1.50) in the follow-up survey of 2021 (see Figure 1). Nineteen percent scored more than or equal to the 5.88-point cutoff at baseline, compared to 18.8% in the follow-up survey (Cutoff value = 5.62). In the cross-sectional sample of 2020, a mean of 4.32 (SD = 1.60) was reported. The cross-sectional sample in 2021 revealed a mean of 4.08 (SD = 1.45). In the cross-sectional samples, 18.8% reached high emotional exhaustion scores (Cutoff value = 5.92) in 2020 and 18.4% (Cutoff value = 5.53) in 2021.

### Prevalence of somatic symptoms and emotional exhaustion differentiated into subgroups of sociodemographic and study related variables

An independent *t*-test revealed that among the cross-sectional sample at T1 non-medical students reported significantly (*p* < 0.001) higher mean scores for somatic symptoms than medical-students. Moreover, a significant (*p* < 0.003) higher mean score of somatic symptoms occurred in 1-year students compared to non-1-year students. Furthermore, there was a significant difference (*p* < 0.001) in mean scores for somatic symptoms between genders, with women achieving higher scores than men. Due to the small group size, participants who indicated themselves as diverse or open (0.7%) were excluded from the independent *t*-tests of somatic symptoms and emotional exhaustion.

Regarding the mean score of emotional exhaustion, no difference between non-medical and medical students was evident. In contrast, significant differences in emotional exhaustion were investigated between subgroups regarding years of enrolment (*p* < 0.001) and gender (*p* < 0.001): 1-year students reached higher mean scores of emotional exhaustion. In addition, women reported higher mean scores of emotional exhaustion than men.

More information regarding subgroup differences in somatic symptoms and emotional exhaustion are provided in Table 2.

**Table 2:** Prevalence of somatic symptoms and emotional exhaustion differentiated into subgroups.

Independent study variables	Somatic symptoms; mean ±*SD*; and its statistical difference between categories of independent variables	Emotional exhaustion; mean ±*SD*; and its statistical difference between categories of independent variables
**Field of study**	t_(2840)_ = 5.24; ***p*** **<** **0.001**; Cohen's *d* = 0.73	t_(2846)_ = 1.23; *p* = 0.22
Non-medical students	1.24 (0.73)	4.34 (1.60)
Medical students	1.01 (0.66)	4.22 (1.57)
**Years of enrolment**	t_(1159.12)_ = −3.00; ***p*** **<** **0.003**; Cohen's *d* = 0.73	t_(1330.98)_ = −4.63; ***p*** **<** **0.001**; Cohen's *d* = 1.59
Non-first-year	1.19 (0.72)	4.25 (1.63)
First-year	1.28 (0.76)	4.56 (1.48)
**Gender**	t_(1489)_ = −12.51; ***p*** **<** **0.001**; Cohen's *d* = 0.71	t_(2879)_ = −4.01; ***p*** **<** **0.001**; Cohen's *d* = 1.59
Male	0.95 (0.65)	4.12 (1.56)
Female	1.30 (0.73)	4.39 (1.61)

### Study-related predictors of somatic symptoms and emotional exhaustion among the longitudinal sample

Correlation analyses (see Supplementary Table 1) revealed that academic self-efficacy, social support from lecturers and peers, workload and work complexity were significantly associated with both somatic symptoms and emotional exhaustion. Therefore, two multiple linear regression analysis were computed with these variables as predictors and somatic symptoms as the dependent variable in the first regression model as well as emotional exhaustion in the second model.

The first linear regression analysis (see Table 3) included *n* = 237 participants. Homoscedasticity and normality of the residuals were confirmed by scatterplot, histogram, and P-P plot. No residual autocorrelation was indicated by a Durbin-Watson statistic of 1.70. An average Variance Inflation Factor (VIF) of 1.40 revealed no collinearity of the selected variables. The overall linear regression model was statistically significant, F(5, 231) = 3.06, *p* ≤ 0.011. The Nagelkerke *R*^2^ further illustrated that the predictors explained 6% of the variation in somatic symptoms. None of the independent variables significantly predicted somatic symptoms. Further information on the linear regression model that includes the autoregressive effect is demonstrated in Table 3.

**Table 3:** Predictors of somatic symptoms at T2 using multiple regression analysis.

Dependent variable: somatic symptoms T2							
**Predictor variable**	***B***	***SE***	**β**	***T***	***p***	**95% CI**	
						***LB***	***UB***
Self-efficacy T1	0.068	0.059	0.079	1.146	0.253	−0.049	0.185
Somatic symptoms T1	0.585	0.053	0.617	11.009	< 0.001	0.480	0.689
Social support: lecturer T1	0.011	0.042	0.016	0.272	0.786	−0.071	0.094
Social support: peers T1	−0.031	0.034	−0.050	−0.907	0.366	−0.097	0.036
Work complexity T1	0.057	0.037	0.103	1.550	0.122	−0.015	0.129
Workload T1	−0.011	0.029	−0.022	−0.371	0.711	−0.068	0.047

*N* = 237; *R*^2^ = 0.386; korr. *R*^2^ = 0.370; F (6,230) = 24.072; *p* < 0.001. *B*, unstandardized estimates; *SE*, standard error; β, standardized estimates; *LL*, lower bound; *UL*, upper bound.

The second linear regression analysis (see Table 4) included *n* = 230 cases. The residuals' homoscedasticity and normality were verified using a P-P plot, histogram, and scatterplot. A Durbin-Watson statistic of 0.46 revealed no autocorrelation of the residuals. With a mean VIF of 1.40, no collinearity of the chosen variables was observed. The overall linear regression model was statistically significant, F(5, 224) = 15.77, *p* ≤ 0.001. The predictors accounted for 26% of the variation in emotional exhaustion (Nagelkerke R^2^). The model revealed that workload (B = 0.20, p < 0.05) and work complexity (B = 0.28, *p* < 0.05) positively predicted emotional exhaustion. A significant negative correlation was demonstrated between self-efficacy and emotional exhaustion (B = −0.41, *p* < 0.05). Including the autoregressive effect, work complexity emerged as the only significant predictor and was shown to be positively related to changes in emotional exhaustion (B = 0.22, *p* < 0.05). Table 4 provides further details on the linear regression model that includes the autoregressive effect.

**Table 4:** Predictors of emotional exhaustion at T2 using multiple regression analysis.

Dependent variable: emotional exhaustion T2							
**Predictor variable**	***B***	***SE***	**β**	***T***	***p***	**95% CI**	
						***LB***	***UB***
Self-efficacy T1	−0.281	0.156	−0.137	−1.796	0.074	−0.589	0.027
Emotional exhaustion T1	0.212	0.074	0.225	2.859	0.005	0.066	0.357
Social support: lecturer T1	−0.153	0.112	−0.088	−1.370	0.172	−0.373	0.067
Social support: peers T1	0.163	0.088	0.112	1.855	0.065	−0.010	0.337
Work complexity T1	0.220	0.098	0.166	2.239	**0.026**	0.026	0.413
Workload T1	0.118	0.080	0.100	1.474	0.142	−0.040	0.275

*N* = 230; *R*^2^ = 0.287; korr. *R*^2^ = 0.267; F(6,223) = 14.925; *p* < 0.001.

*B*, unstandardized estimates; *SE*, standard error; β, standardized estimates; *LB*, lower bound; *UB*, upper bound.

## Discussion

The results of the present study demonstrate that approximately one-third of all students exhibited critical somatic symptom scores, exceeding the 12-point cutoff in both the longitudinal and cross-sectional samples. A prevalence of 18%−19% of high emotional exhaustion scores indicates that almost one in five students experienced high levels of burnout. From 2020 to 2021, a slight decrease in somatic symptoms and emotional exhaustion was noted. Regarding subgroup differences, this study indicates that non-medical students reached higher mean scores of somatic symptoms compared to medical students. Additionally, the results highlight particular vulnerabilities among women and 1-year students for both somatic symptoms and emotional exhaustion. Furthermore, longitudinal analyses indicate that emotional exhaustion increased with higher perceived work complexity.

Addressing the first research aim of investigating the prevalence and development of somatic symptoms and emotional exhaustion among university students from 2020 to 2021, the results revealed similar mean scores and prevalences of somatic symptoms between the cross-sectional and longitudinal samples. The prevalence rates in the cross-sectional samples were of interest due to the large sample sizes and were therefore included. The mean score of 1.20 in the longitudinal sample in 2020 decreased by 0.08 points in 2021. It is particularly striking that approximately one in three students reported being highly affected by somatic symptoms at both time points across all samples. This is consistent with the reported high prevalence rate of somatic symptoms among medicals students of 26 % in 16 countries worldwide (13), and highlights the high prevalence of somatic symptoms among all university students. Considering the high prevalence and association with severe impairment and psychological distress (12, 14, 15, 44, 45), somatic symptoms, whether they can be medically explained or not, should be acknowledged as a prominent concern among university students and require greater attention in future research.

As mentioned above, this study also investigated the prevalence and development of emotional exhaustion. The mean scores and prevalences of emotional exhaustion were closely similar across the cross-sectional and longitudinal samples. The mean score of 4.31 in the longitudinal sample in 2020 decreased to 4.12 in 2021. Previous studies also using the Maslach Burnout Inventory for students reported mean scores from 2.10 in 2008 (64) to 3.85 in 2019 and 4.17 in 2020 (65). The results of the present study align with the observed upward trend in emotional exhaustion scores over time, indicating a concerning increase in emotional exhaustion among students. These rising levels suggest an increased risk of burnout among university students, since emotional exhaustion is frequently reported as its initial stage (8). This study adds further evidence to the previously reported high prevalence rates of emotional exhaustion among university students (10) by revealing that 18%−19% exceeded the cutoff value, indicating high levels of burnout. The prevalence rates observed in this study are far above those observed by Obregon et al. (2020) (11), with 8% of the participants scoring at least one SD above the mean. This interesting difference might be explained by the fact that the above-mentioned study was conducted among medical students. Therefore, it can be suggested that emotional exhaustion is a general problem in universities and not exclusively among medical students.

Despite a noted decrease from 2020 to 2021, mean scores for emotional exhaustion and somatic symptoms remained higher across all samples compared to pre-pandemic levels reported in a previous study of our group. Regarding somatic symptoms, Reichel et al. (2023) (65) demonstrated a mean score of 1.12 before the pandemic and observed no significant change during the pandemic from 2019 to 2020, also using the SSS-8 to asses somatic symptoms. In terms of emotional exhaustion, Reichel et al. (2023) (65) reported that the mean score increased significantly from 3.85 before the pandemic to 4.17 in 2020. There are several possible explanations for the decrease from 2020 to 2021, despite the overall higher scores compared to pre-pandemic levels. One explanation could be that Germany eased its COVID-19 restrictions in 2021 (66), therefore students could have experienced less pandemic-related stress. Sun et al. (2022) (39) provide an additional explanation, stating that during the early stages of COVID-19, the unexpected circumstances caused high levels of stress and psychological symptoms in the majority of people, but over the time resilience helped them adapt resulting in decreasing stress levels. The findings of the present study, along with previous research, indicate that high levels of somatic symptoms and emotional exhaustion are prevalent regardless of the pandemic. This emphasizes the importance of interventions to lower baseline levels and providing students with effective coping strategies to better manage future crises.

In view of the second research aim, which is to identify subgroup differences in emotional exhaustion and somatic symptoms, the results revealed that non-medical students exhibited higher mean scores of somatic symptoms compared to medical students. Therefore, the present study questions the overemphasis on medical students in the existing literature. Additionally, no significant differences between medical and non-medical students were found in terms of emotional exhaustion. However, mean scores from non-medical students were slightly higher than those of medical students. The results challenge the common assumption that medical students might be particularly vulnerable to somatic symptoms and emotional exhaustion compared to other student groups due to stressors attributed specifically to medical training, such as a demanding and time-intensive workload, conflicts with work-life-balance, a competitive environment and a frequent contact to illness, suffering and death (23, 67–69). The present study highlights a critical gap in the current understanding, suggesting that students face individual challenges in each academic field, which require further investigations regarding the prevalence, predictors and etiology of somatic symptoms and emotional exhaustion across different study fields.

Furthermore, females reported significantly higher mean scores for somatic symptoms and emotional exhaustion than males. Therefore, the present study further verifies the assumption of previous research worldwide that female gender appears as a risk factor for somatic symptoms (12, 22, 23, 28, 41, 48). The current state of research is inconsistent regarding gender differences in emotional exhaustion. The results of a study conducted in 2021 (34) are in line with the present results, suggesting females might experience higher levels of emotional exhaustion than males. A study performed in earlier stages of the COVID-19 pandemic (2019–2020) (7) supports evidence from previous research before the pandemic started (10), proposing no gender difference in emotional exhaustion. It seems possible that the results regarding gender differences were influenced by long-term effects of the pandemic, as females and males were differently affected (70). Each subgroup experienced unique stressors (70, 71), which may have influenced the levels of emotional exhaustion in females and males in different ways. Consequently, the results could not be generalized and must be interpreted with caution.

Moreover, mean scores for somatic symptoms and emotional exhaustion were significantly higher among first-year students than among students in later stages of their studies. Very little was found in the literature on whether students are most vulnerable to somatic symptoms and emotional exhaustion in their first year of studies or if these symptoms develop progressively over the years at university. Recently, a higher prevalence of musculoskeletal pain among medical students in their clinical years compared to the preclinical years was suggested (72). It is important to bear in mind that the structure of medical school is different across various countries, which could influence the manifestation of somatic symptoms (13) and not only medical school but also each study field differs in their structure and demands. Moreover, previous research proposed that the prevalence rates of somatic symptoms vary depending on the type of symptom (73). Therefore, further research is suggested to explore the development of different types of somatic symptoms over the time at university across different study fields.

Regarding emotional exhaustion, Olson et al. (2023) (7) questioned the previous assumption that a higher number of semesters would be associated with an increase (34). Our results further support the postulation of Olson et al. (2023) (7) that a successful study-progression could rather lead to a decrease of emotional exhaustion. The results of the present study further indicate that students in their first year of university were more vulnerable to somatic symptoms and emotional exhaustion than their further progressed peers, since these students experience challenging life changes during the first year commencing university such as moving away from family and the development of a new social network (74). Since the 1-year students of the present study started their studies during the COVID-19 pandemic, it is possible that pre-existing challenges could have been amplified by pandemic-related restrictions. It is crucial to highlight that even among the subgroups of students less affected, particularly the mean scores for emotional exhaustion were concerningly high, which emphasizes the need for interventions for all student groups.

Regarding the third research aim, regression analysis was used to investigate potential study-related predictors of somatic symptoms and emotional exhaustion among students. Among the factors facilitating emotional exhaustion, academic self-efficacy was shown to be a negative predictor of emotional exhaustion, which is consistent with the literature (33, 65). In line with previous studies, workload demonstrated as a positive predictor of emotional exhaustion (9, 65, 75). Including the autoregressive effect, only work complexity emerged as a significant predictor and was revealed to be positively related to changes in emotional exhaustion. This confirms previous research postulating a strong relationship between work complexity and emotional exhaustion (65). A possible explanation for why academic self-efficacy and workload did not remain significant predictors after including the autoregressive effect could be that a 1-year timespan between the two measurement points in the longitudinal sample could be too long to accurately assess more proximal predictors of emotional exhaustion (65). This explanation indicates that academic self-efficacy and workload rather have a short-term effect on emotional exhaustion, whereas high work complexity leads to an increase in emotional exhaustion over time. Contrary to the findings of Reichel et al. (2023) (65), this study did not reveal social support from peers and lecturers as a significant predictor. As discussed earlier, these differences could be explained by the fact that the mentioned study was conducted in the earlier phases of the COVID-19 pandemic whereas the present study was performed in the later stages with different levels of social restrictions, making it challenging to compare these timespans regarding social networks. Therefore, it remains unclear to which degree the social support experienced in university contributes to changes in emotional exhaustion.

In contrast to earlier studies, which have suggested academic self-efficacy and social support from lecturers as significant predictors for somatic symptoms (47, 65), none of the examined independent variables predicted somatic symptoms. Our results can be explained by several hypotheses of the current state of research. In line with the ideas of Feussner et al. (2022) (12), it could be concluded that multiple factors interact to influence somatic symptoms rather than a single factor being responsible. Following the hypothesis of Reichel et al. (2023) (65) our results further support the idea that stressors initially affect emotional exhaustion as a proximal indicator whereas the prolonged exposure to these stressors influences distal health indicators such as somatic symptoms. Another explanation could be that stressors, such as work complexity, rather have an indirect impact on somatic symptoms by increasing psychological distress, such as emotional exhaustion, which in turn could contribute to the development of somatic symptoms (16, 17). Further research is needed to explore the interaction of emotional exhaustion and somatic symptoms.

The present study has limitations. The study sample's composition was different from that of the entire student body, with a lower mean age, higher proportion of females and a possibly higher participation rate of students who are interested in health-related topics. Moreover, only one university in Germany was included in this study making the results less generalizable to other universities and possibly not reproducible on a wide scale across different countries. The results may be biased since the acquired data was self-reported. For example, it was demonstrated that emotional exhaustion may be more closely associated with subjective than objective workload (9). Therefore, objective workload may be less likely to predict emotional exhaustion.

## Conclusion with implications for further research and focus on practical recommendations

To draw an overall conclusion: the prevalence rates of somatic symptoms and emotional exhaustion among university students were concerningly high. Despite being understudied, somatic symptoms appeared to be a prominent problem in university education, emphasizing the need for further investigations regarding the predictors and etiology. The present study questions the prevailing focus on medical students in terms of emotional exhaustion and somatic symptoms and highlights the need for further research across different study fields. Through determining individual stressors and challenges attributed to each academic field, targeted interventions could be established.

Already with the start of study, students should be educated about the importance and presence of health-promoting programs as well as the possible connection between physical and psychological distress. As a result, not only could students practice and adopt health-promoting habits from early on and strengthen these over the years at university, but it would also be easier for struggling students to reach out for help. Practical recommendations could include the implementation of online courses accessible to everyone for learning basic skills in time and stress management. Additionally, students with specific struggles could benefit from problem-focused courses, for example overcoming self-doubt or exam anxiety. One example of this approach is the Health Express as part of the Healthy Campus Mainz project. Lecturers could contribute to health-promoting education of students by including short videos about health and study management in their lectures. Moreover, the Healthy Campus Mainz project offered students online courses targeting various study-related issues. Another example is the Mental Health Services of Johannes Gutenberg University Mainz, which offers students help in pressing crises, counseling, short-term psychotherapy, and workshops. To better support 1-year students during upcoming life changes and challenges, opportunities for networking with more advanced peers could be enhanced. Therefore, 1-year students could benefit from their peers' experience in terms of organization and learning strategies tailored to their field of study.

The mental and physical health of university students is not just of interest to the students themselves, but to our whole society. Universities could distinguish themselves by developing graduates equipped with key skills, empowering them to meet challenges with excitement, to identify and manage problems with a solution-oriented approach and consequently to contribute as leaders of tomorrow to an efficient and resilient society.

## Data Availability

The raw data supporting the conclusions of this article will be made available by the authors, without undue reservation.
