# Supplementary material for: Predictor and subgroup analysis of somatic symptoms and emotional exhaustion among university students in Germany during the SARS-CoV-2 pandemic: a longitudinal analysis
Source: Front Public Health. 2026 May 13;14:1827225. doi: 10.3389/fpubh.2026.1827225 (PMC13212139; doi:10.3389/fpubh.2026.1827225)
Supplement: Supplementary file 1 [file Table_1.docx]

**Supplemental Table 1.** Bivariate correlations between study variables.

| Variable | 1 | 2 | 3 | 4 | 5 | 6 | 7 | 8 | 9 | 10 | 11 | 12 | 13 | 14 |
| --- | --- | --- | --- | --- | --- | --- | --- | --- | --- | --- | --- | --- | --- | --- |
| 1. Somatic symptoms T2 | - | 0.603^**^ | 0.286^**^ | 0.396^**^ | -0.102 | -0.127 | -0.069 | -0.203^**^ | 0.140^*^ | 0.205^**^ | -0.049 | -0.082 | -0.002 | -0.143^*^ |
| 2. Somatic symptoms T1 | 0.603^**^ | - | 0.475^**^ | 0.300^**^ | -0.186^**^ | -0.159^*^ | -0.178^**^ | -0.356^**^ | 0.253^**^ | 0.265^**^ | -0.053 | -0.023 | -0.012 | -0.133^*^ |
| 3. Emotional exhaustion T1 | 0.286^**^ | 0.475^**^ | - | 0.446^**^ | -0.417^**^ | -0.223^**^ | -0.436^**^ | -0.560^**^ | 0.547^**^ | 0.550^**^ | -0.092 | -0.028 | 0.085 | -0.003 |
| 4. Emotional exhaustion T2 | 0.396^**^ | 0.300^**^ | 0.446^**^ | - | -0.283^**^ | -0.019 | -0.247^**^ | -0.396^**^ | 0.353^**^ | 0.429^**^ | -0.144^*^ | -0.118 | 0.111 | 0.097 |
| 5. Social support: lecturer (T1) | -0.102 | -0.186^**^ | -0.417^**^ | -0.283^**^ | - | 0.263^**^ | 0.463^**^ | 0.368^**^ | -0.281^**^ | -0.328^**^ | 0.019 | 0.034 | -0.009 | -0.110 |
| 6. Social support: peers (T1) | -0.127 | -0.159^*^ | -0.223^**^ | -0.019 | 0.263^**^ | - | 0.152^*^ | 0.277^**^ | -0.117 | -0.077 | -0.062 | -0.079 | 0.058 | 0.150^*^ |
| 7. Autonomy (T1) | -0.069 | -0.178^**^ | -0.436^**^ | -0.247^**^ | 0.463^**^ | 0.152^*^ | - | 0.344^**^ | -0.332^**^ | -0.316^**^ | 0.093 | -0.015 | -0.034 | -0.356^**^ |
| 8. Academic self-efficacy (T1) | -0.203^**^ | -0.356^**^ | -0.560^**^ | -0.396^**^ | 0.368^**^ | 0.277^**^ | 0.344^**^ | - | -0.355^**^ | -0.538^**^ | 0.189^**^ | 0.071 | 0.079 | 0.003 |
| 9. Workload (T1) | 0.140^*^ | 0.253^**^ | 0.547^**^ | 0.353^**^ | -0.281^**^ | -0.117 | -0.332^**^ | -0.355^**^ | - | 0.453^**^ | -0.124^*^ | -0.013 | 0.112 | 0.073 |
| 10. Work complexity (T1) | 0.205^**^ | 0.265^**^ | 0.550^**^ | 0.429^**^ | -0.328^**^ | -0.077 | -0.316^**^ | -0.538^**^ | 0.453^**^ | - | -0.170^**^ | -0.056 | 0.033 | 0.023 |
| 11. Semester (T1) | -0.049 | -0.053 | -0.092 | -0.144^*^ | 0.019 | -0.062 | 0.093 | 0.189^**^ | -0.124^*^ | -0.170^**^ | - | 0.597^**^ | -0.032 | 0.212^**^ |
| 12. Age (T1) | -0.082 | -0.023 | -0.028 | -0.118 | 0.034 | -0.079 | -0.015 | 0.071 | -0.013 | -0.056 | 0.597^**^ | - | -0.147^*^ | 0.342^**^ |
| 13. Field of study (T1) | -0.002 | -0.012 | 0.085 | 0.111 | -0.009 | 0.058 | -0.034 | 0.079 | 0.112 | 0.033 | -0.032 | -0.147^*^ | - | -0.063 |
| 14. Aspired degree (T1) | -0.143^*^ | -0.133^*^ | -0.003 | 0.097 | -0.110 | 0.150^*^ | -0.356^**^ | 0.003 | 0.073 | 0.023 | 0.212^**^ | 0.342^**^ | -0.063 | - |
| *Note.* * p < .05. ** p < .01. | | | | |  |  |  |  |  |  |  |  |  |  |

**Supplemental Table 2.** CFA results for used items.

|  | χ^2^ | df | CFI | TLI | RMSEA | SRMR |
| --- | --- | --- | --- | --- | --- | --- |
| T1 | | | | | | |
| 1 Factor | 1843.40 | 81 | .54 | .50 | .14 | .11 |
| 2 Factors | 1617.37 | 82 | .61 | .58 | .13 | .10 |
| 4 Factors | 1218.79 | 87 | .73 | .70 | .11 | .09 |
| 5 Factors | 1056.27 | 91 | .78 | .75 | .10 | .08 |
| T2 | | | | | | |
| 1 Factor | 2073.78 | 81 | .51 | .47 | .15 | .12 |
| 2 Factors | 1722.94 | 82 | .61 | .58 | .13 | .11 |
| 4 Factors | 1221.65 | 87 | .75 | .72 | .11 | .10 |
| 5 Factors | 998.06 | 91 | .81 | .79 | .09 | .09 |
